# A retrospective study about functional outcome and quality of life after surgical fixation of insufficiency pelvic ring injuries

**DOI:** 10.1186/s12891-021-04925-y

**Published:** 2021-12-13

**Authors:** Katharina Jäckle, Marc-Pascal Meier, Mark-Tilmann Seitz, Sebastian Höller, Christopher Spering, Mehool R. Acharya, Wolfgang Lehmann

**Affiliations:** 1 Department for Trauma Surgery, Orthopaedics and Plastic Surgery, University Medical Center Göttingen, Robert-Koch Str. 40, 37075 Göttingen, Germany; 2 Pelvic and Acetabular Reconstruction Unit, Department of Trauma & Orthopaedics, North Bristol NHS Trust, Southmead Hospital, Southmead Rd, Bristol, BS10 5NB UK

**Keywords:** Fragility fractures, Percutaneous screw fixation, Long-term outcome, Quality of life

## Abstract

**Background:**

Fragility fractures without significant trauma of the pelvic ring in older patients were often treated conservatively. An alternative treatment is surgery involving percutaneous screw fixation to stabilize the posterior pelvic ring. This surgical treatment enables patients to be mobilized quickly and complications associated with bedrest and temporary immobility are reduced. However, the functional outcome following surgery and quality of life of the patients have not yet been investigated. Here, we present a comprehensive study addressing the long-term well-being and the quality of life of patients with fragility pelvic ring fractures after surgical treatment.

**Methods:**

Between 2011–2019, 215 geriatric patients with pelvic ring fractures were surgically treated at the university hospital in Göttingen (Germany). Of these, 94 patients had fragility fractures for which complete sets of computer tomography (CT) and radiological images were available. Fractures were classified according to Tile and according to the FFP classification of Rommens and Hofmann. The functional outcome of surgical treatment was evaluated using the Majeed pelvic score and the Short Form Health Survey-36 (SF-36).

**Results:**

Thirty five tile type C and 48 type B classified patients were included in the study. After surgery eighty-three patients scored in average 85.92 points (± 23.39) of a maximum of 100 points using the Majeed score questionnaire and a mean of 1.60 points on the numerical rating scale ranging between 0 and 10 points where 0 points refers to “no pain” and 10 means “strongest pain”. Also, the SF-36 survey shows that surgical treatment positively effects patients with respect to their general health status and by restoring vitality, reducing bodily pain and an increase of their general mental health.

**Conclusions:**

Patients who received a percutaneous screw fixation of fragility fractures of the posterior pelvic ring reported an overall positive outcome concerning their long-term well-being. In particular, older patients appear to benefit from surgical treatment.

**Trial registration:**

Functional outcome and quality of life after surgical treatment of fragility fractures of the posterior pelvic ring, DRKS00024768. Registered 8th March 2021 - Retrospectively registered. Trial registration number DRKS00024768.

## Background

Pelvic ring injuries are relatively rare injuries [1, 2], accounting for only about 3% of all fractures. However, polytraumatized patients were diagnosed with pelvic ring injuries in almost 25% of cases [1, 2]. Furthermore, in older patients pelvic injuries occur frequently [1, 3]. A reason is the low-energy trauma mechanism, which leads to fractures in bone with a lower bone density [1]. Such fractures, referred to as fragility fractures, could be both a symptom or a sign of osteoporosis. The incidence of pelvic ring fractures in older people is 90/100,000 and it is increasing due to demographic changes [3], as reflected by the steadily increasing proportion of older people in any population, leading generally to an aging population (for details see reference [3]). Fragility fractures, are traditionally treated conservatively [3]. The conservative treatment includes optimization of analgesia and pain-adapted mobilization. However, this conservative treatment is associated with potentially severe mid-term and long-term complications [4] including a high mortality rate, i.e. the mortality rate within one year after the fracture is reported to be 19% and even higher (27%) when patients lived on their own or in nursing homes after the fractured pelvis [3].

Pelvic injuries including fragility fractures can be distinguished as described by Tile [5] and classified according to Young and Burgess [6] and Rommens and Hofmann [7], respectively. The injuries can occur in multiple different ways. Among those, pure sacroiliac fractures or transiliacal fractures are much rarer in fragility fractures than lateral sacral fractures close to the iliosacral joint. Very little has been reported on the midterm clinical and functional outcome of surgical treatment in this population. In the present study, we wanted to investigate the clinical and functional outcome after surgical treatment of fragility fractures of the pelvis to determine whether these patients benefit from surgical care.

## Methods

### Patient collective

**Fig. 1:**
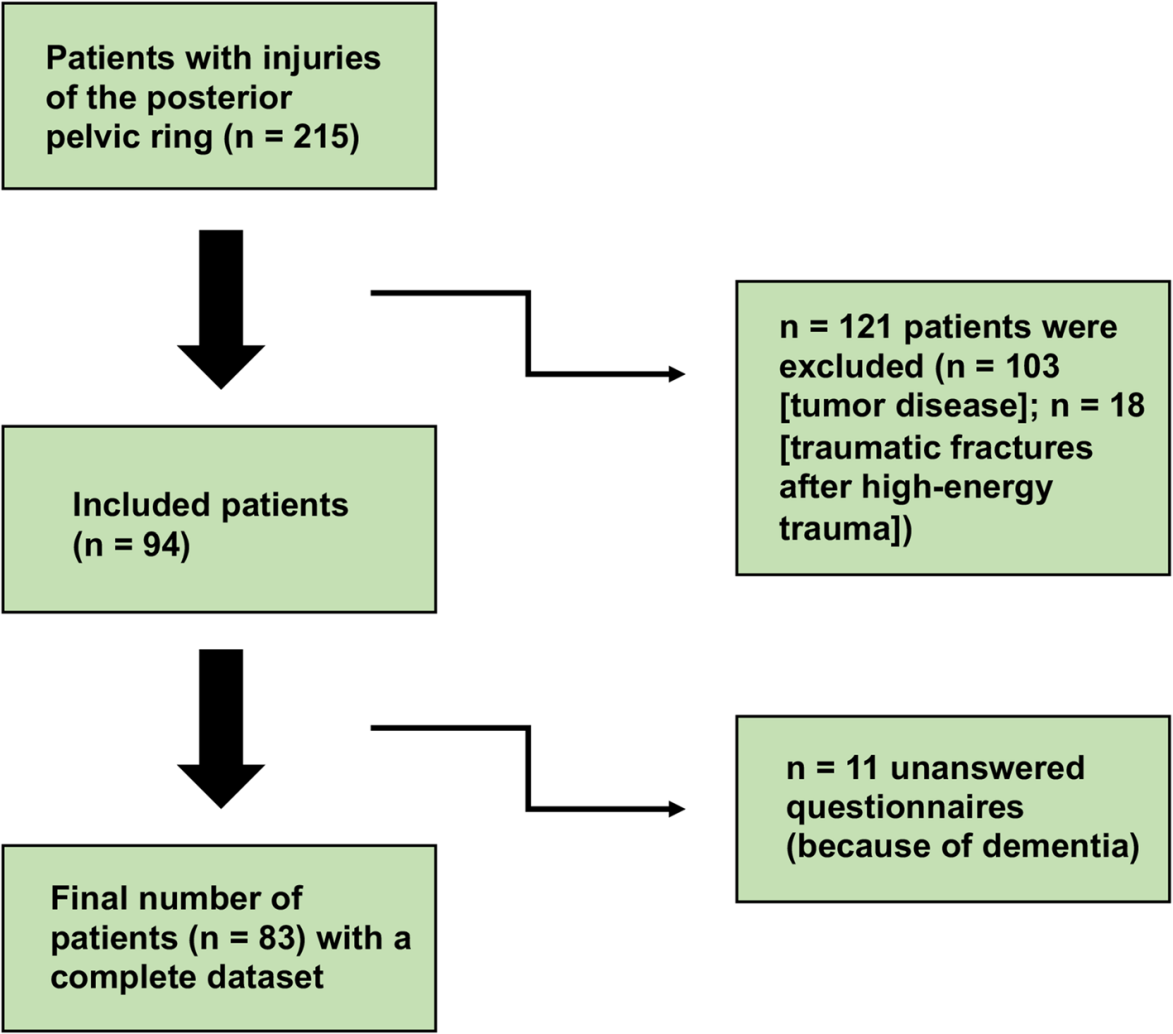
Patient inclusion and exclusion criteria. Flowchart of the inclusion and exclusion algorithm

The present study was approved by the Ethics Committee of the University Medical Center Göttingen (approval number: AN 35/10/20). All patients with a fragility fracture of the posterior pelvic ring, who were admitted for surgery at the University Hospital of Göttingen between 2011 and 2019, were included in a retrospective study. Out of a total of 215 geriatric patients with injuries of the posterior pelvic ring, who were surgically treated during this period, 94 patients with fragility fractures of the posterior pelvic ring (mean age: 72.70 ± 7.86; 59 females and 35 males) who had previously presented in the emergency department were included in the study. Pelvic injuries were assessed both preoperatively and postoperatively using CT diagnostics and X-ray images. Preoperatively, the injuries were classified independently by two experienced pelvic surgeons according the Tile classification [5] and the FFP classification according to Rommens and Hofmann [7] (Table 1). Furthermore, images which were taken after the surgery were examined independently by two experienced pelvic surgeons to determine the precise location of the implant position. Exclusion criteria for the study were follow up examinations over a period of less than one year after the surgery and patients with a history of tumor disease or traumatic fractures (see Fig. 1).

**Table 1:** Classification of fragility fractures according to Rommens and Hofmann

FFP-type	Description	n
1: *Anterior pelvic ring injury only*	a: isolated unilateral anterior disruption	–
	b: isolated bilateral anterior disruption	–
2: *Non-displaced posterior injury*	a: isolated, non-displaced sacral fracture without involvement of the anterior pelvic ring	28
	b: non-displaced sacral crush with anterior disruption	3
	c: non-displaced sacral, iliosacral or ilium fracture with anterior disruption	2
3: *Displaced unilateral posterior injury*	a: displaced unilateral iliac fracture	3
	b: displaced unilateral iliosacral disruption	10
	c: displaced unilateral sacral fracture	2
4: *Displaced bilateral posterior injury*	a: bilateral iliac fracture or bilateral iliosacral disruption	17
	b: bilateral sacral fracture, spinopelvic dissociation	3
	c: combination of different dorsal instabilities	15

*FFP* fragility fractures of the pelvis

### Surgical intervention

Patients were selected on the basis of operations and procedures key (OPS) coding (5-79a.0e; 5-79b.0e; 5–790.0d; 5–798.3) and international classification of diseases (ICD)-10 coding (S32.1; S32.89). All patients of the study had fragility fractures of the posterior pelvic ring, and stabilization was achieved by percutaneous fixation (see Table 2). Beside other stabilization methods percutaneous screw fixation is one of the most frequently used operation techniques. For this treatment, one or two cannulated screws were inserted at the level of S1 and/or S2 into the sacrum, controlled by radiology [8]. Cannulated screws with thread diameter between 7.3 and 7.5 mm and a length between 75 and 110 mm with a washer were used (Axomed GmbH, Freiburg, Germany). The decision to treat fractures with one or two sacroiliac screws always had to be made individually, i.e. depending on the particular injury of the patient that could be classified as according to the FFP classification which distinguishes between different levels of instability (see Table 1 below). The use of two sacroiliac screws achieves greater stability, so this procedure was used for unstable fractures. The anterior pelvic ring was additionally stabilized by implanting an external fixator if the fracture remained unstable (n = 9).

**Table 2:** Gender distribution and baseline characteristics of the population

Number of female patients	51
Percent of female patients [%]	61.45
Number of male patients	32
Percent of male patients [%]	38.55
Ratio (males/females)	0.63
Age range [years]	60–87
Age mean [years] ± SD	71.90 ± 7.69
Stationary stay range [days]	2–53
Stationary stay mean [days] ± SD	16.87 ± 10.28

### Follow up

Patients were invited for interviews, providing a precasted questionnaire which was based on the Majeed Pelvic and SF-36 score [9, 10] as described below. The well-being of the respondents was assessed by these interviews which took place in 2020, i.e. at least one year after the last CT scans. CT diagnostics were performed both preoperatively and immediately postoperatively. Of the 94 patients who were contacted for the study, 83 (88.3%) responded with a completed questionnaire. The collected data were evaluated anonymously. The 11 unanswered questionnaires concerned patients who according to their relatives had dementia. Thus, it was not possible to interview them. Complete data was available for these 83 patients (mean age: 71.90 ± 7.69; 51 females and 32 males; see Table 2). They included 35 Tile type C and 48 type B injuries, which were also classified according to classification of fragility fractures according to Rommens and Hofmann [7]. Details are described in Table 1. The classification of the pelvic ring fractures was independently performed by two experienced pelvic surgeons. We note that fundamental differences exist between type B and C injuries [5, 6], but it had no effect on the results of their surgical treatment (*p* = 0.052). We combined therefore the B and C types and referred to them collectively as pelvic injuries.

**Fig. 2:**
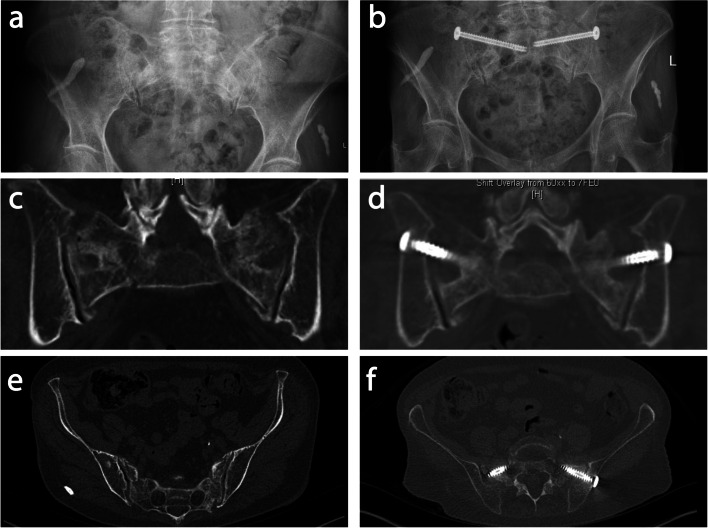
X-ray and CT pictures of one patient showing a bilateral pelvic injury before and after the surgery. **a** X-ray picture, shows a rather inconspicuous finding, before and (**b**) after the surgery. **c** Additional CT-diagnostics (coronar view) clearly shows an injured pelvis (FFP 4b classification) of a patient before and (**d**) after the surgery. **e** CT picture of the same patient before and (**f**) after the surgery in axial view

Shortly after the injury and admission to the hospital, an attempt was made to mobilize the patients. In the instances where patients were unable to mobilize due to pain, surgical treatment was offered. The decision to opt for surgical treatment was usually of 3–5 days after a trial of mobilization. These patients underwent a closed reduction of the posterior pelvic ring and percutaneous sacroiliac screw osteosynthesis, i.e. under radiological control one or two cannulated screws were inserted into the sacrum at the level of S1 and/or S2 (see Table 2). X-ray controls were performed prior to and two days after the surgery as well as three and six weeks before the rehabilitation phase started. They included an antero-posterior view of the pelvis with additional inlet/outlet projections. To determine the proper position of the screw osteosynthesis, additional CT scans were taken both prior to and after the surgery (see Fig. 2). After surgical treatment, patients were mobilized with the support of physiotherapists. The patients were able to put full weight on the affected side in a pain-adapted manner. They were then transferred to a rehabilitation facility for further mobilization therapies.

### Questionnaire and its evaluation

Patients were contacted by letter which included a questionnaire which was based on the Majeed Pelvic score and the SF-36 (version 1.0) [9, 10] and included a numerical rating scale (NRS) to ask about the tenderness over the posterior pelvic ring of the patients.

The Majeed score includes aspects of work conditions and sexuality. These aspects of life and well-being are difficult to evaluate in older patients since the majority of them are retired. However, since the age limit of our patients’ collective started at 60 years, the majority is still at work and older patients are despite their official retirement continued with voluntary work. Thus, this aspect was left in the questionnaire without further qualification. In cases where patients did not refer to their work situation, we scored the corresponding value as “0”, implying that the patients have already retired from work. Accordingly, a “0” was scored if no information was provided on the aspect “sexuality”.

The NRS represents a one-dimensional metric scale to subjectively qualify the pain intensity within the range of zero (no pain) to 10 (strong pain) (see Table 4). Patients were also asked whether they were unsupported in their daily life before injury and after surgery (see Table 4). The interviews were in 2020, i.e. at least one year after obtaining the last CT scans. Thus, for some patients, the interview occurred several (1 up to 9) years after the surgical treatment, the median delay between the trauma and the questionnaire was minimum 52 weeks and maximum 388 weeks.

### Statistical analysis

Normal distribution analysis was performed using the D’Agostino-Pearson test. Significance calculations were based on the Wilcoxon-Mann-Whitney test and the significance level was set to alpha = 5%. Additionally, Pearson correlation test was performed and the confidence interval was set to 95%. Statistics software Graphpad Prism 8 (version 8.1.1 for mac) was used.

## Results

**Fig. 3:**
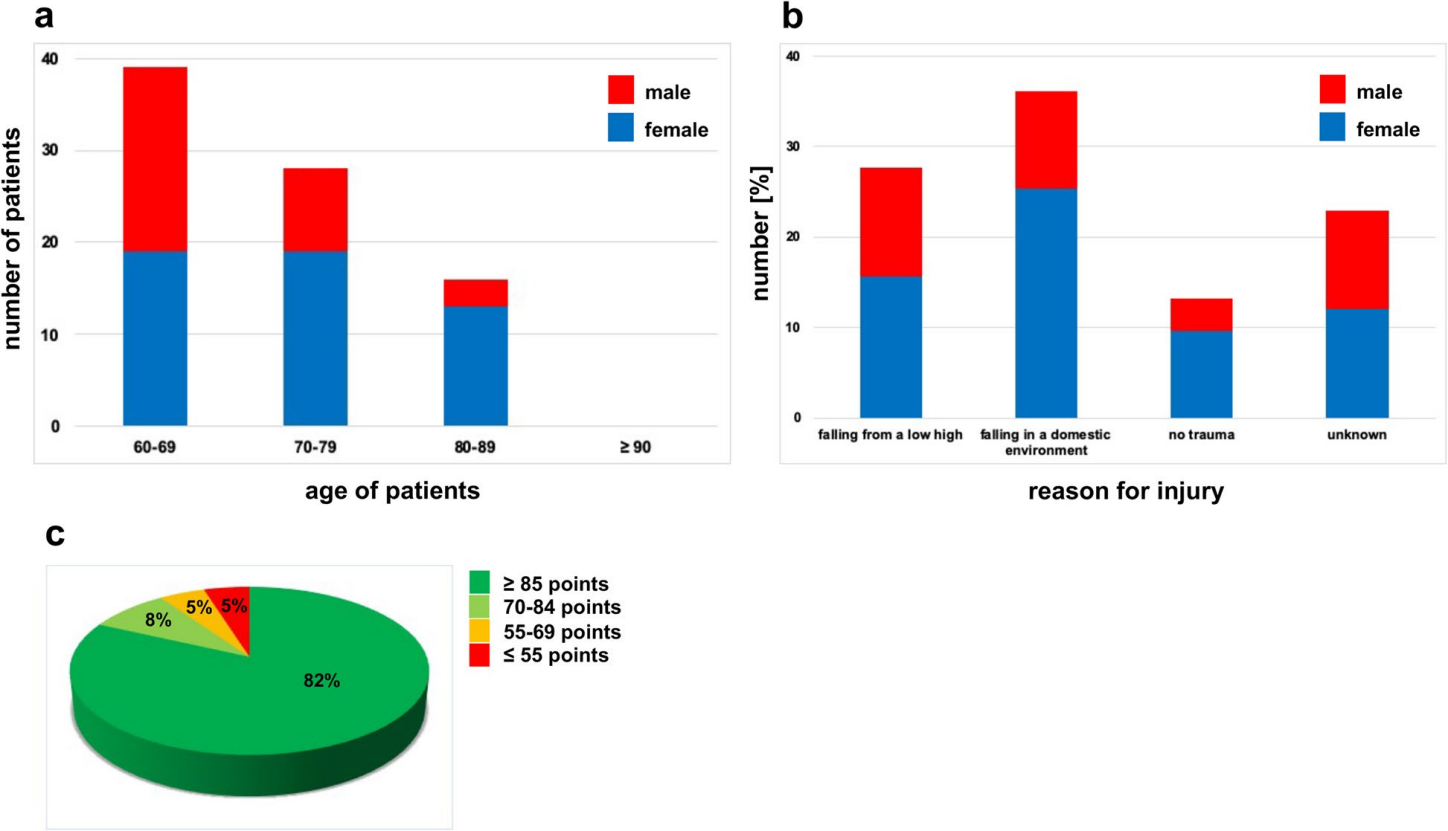
Age distribution, reason for trauma and results of the Majeed score questionnaire in the overview. **a** Age distribution of the patient collective after surgical treatment (n = 83); blue bars show female (n = 51) and red bars male (n = 32) distribution. Median values are shown. **b** Reason for trauma of the patient collective with numbers in % for females (blue bars; n = 51) and males (red bars; n = 32). Median values are shown. **c** Results of Majeed score questionnaire; dark green = excellent, light green = good, orange = fair, red = poor

Ninety-four patients (mean age: 72.70 years (range 60–87 years); 59 females and 35 males) with fragility fractures of the posterior pelvic ring were included in the study. Of those, 83 patients (88%) returned the completed questionnaire. Figure 3a shows the age and gender distribution of the 83 patients who participated in the study.

Figure 3b and Table 3 show the cause of the injuries of the participating patients. The primary cause of the injury was a fall in the domestic environment (36.14%) such as tripping over the edge of a carpet, symptoms of vertigo and gait insecurity. The second major causes were injuries associated with a fall from low height (27.71%), i.e. less than 0.5 m (falling out of bed, fall from a standing height because of symptoms of vertigo). There was no specific history of trauma in 13.25% of cases. In 22.89% of the cases, the cause of the injury was not reported (Table 3; Fig. 3b).

**Table 3:** Causes of injuries of the population

Falling from a low high (n = 23) [%]	27.71
Falling in a domestic environment (n = 30) [%]	36.14
No trauma (n = 11) [%]	13.25
Unknown (n = 19) [%]	22.89

### Evaluation of the well-being of the patients

Table 5 and Fig. 3c show the results of the total Majeed score for all patients with percutaneous insertion of sacroiliac screws. Patients had an average Majeed score of 85.92 (± 23.39) after surgery. Excellent Majeed scores, and thus excellent function, were found with the majority of 68 patients, seven patients had a good outcome and four patients scored either a fair or even a poor outcome of the treatment.

**Fig. 4:**
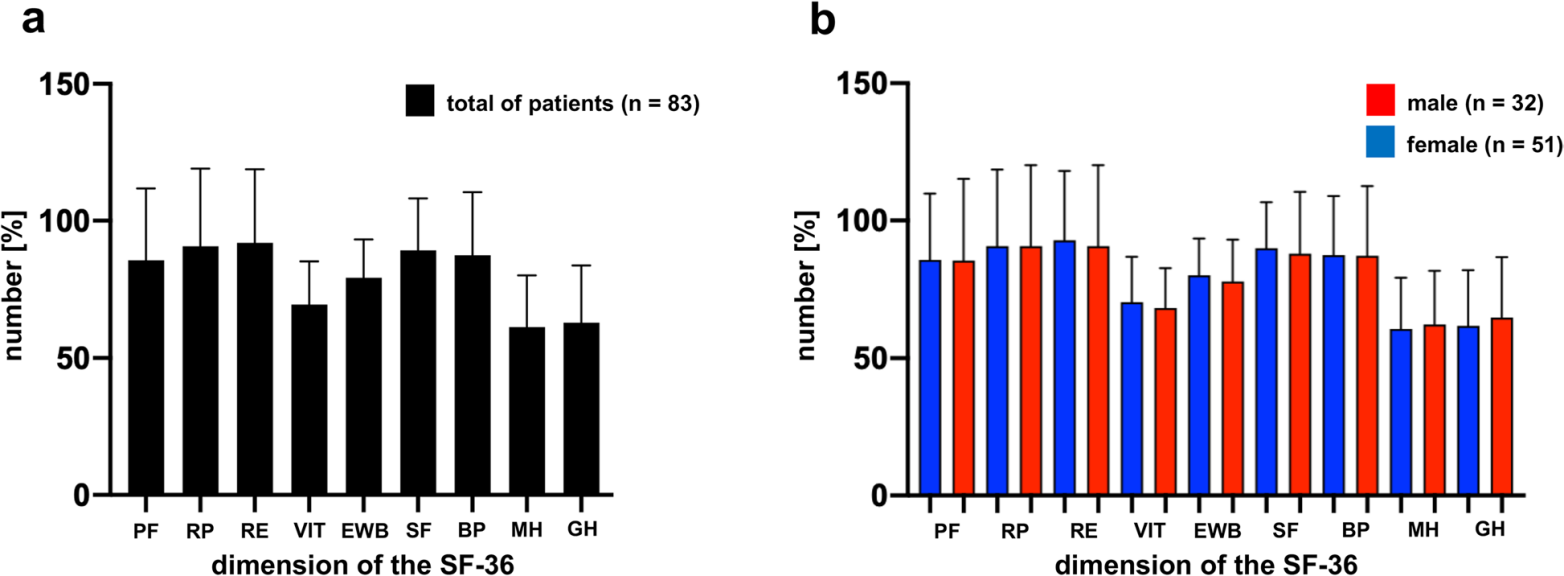
Results of the SF-36 score questionnaire. **a** Results of the SF-36 score questionnaire of the whole patients collective (black bars; n = 83). **b** Results of the SF-36 score questionnaire of the patients collective divided into female (blue bars; n = 51) and male (red bars; n = 32) distribution. On the x-axis the different dimensions were applied. PF = physical functioning; RP = role limitations due to physical health problems; RE = role limitations due to personal or emotional problems; VIT = social functioning, energy/fatigue or vitality; EWB = emotional well-being; SF = social functioning; BP = bodily pain; MH = general mental health; GH = general health perceptions. On the y-axis numbers are shown in %. Error bars show standard deviation. Median values are shown

The individual parameters of the SF-36 are summarized in Table 6. It shows that the scores for all queried aspects concerning the well-being of the patients at least one year after the surgical treatment were rather high (above 60%; range: 61–93%) and no significant gender difference was observed (Table 6 and Fig. 4﻿). Furthermore, there was no significant difference with respect to the scores between patients with different FFP classifications, i.e. 75% of patients with FFP 2, 73% with FFP 3, and 71% of patients with FFP 4 classification showed scores above 85% in the SF-36 score.

The results based on the numerical rating scale (NRS) point out a low indication of pain of the patients after surgical treatment. The mean NRS value of 1.60 points was scored by the patients that had a surgical intervention. The results are detailed in Table 4.

**Table 4:** NRS results and distribution of independence for the population

NRS [points]	1.60 ± 2.14
Unsupported living before injury and after surgery [n]	81
Supported living before injury and after surgery [n]	2

The vast majority of the patients (81 of the 83 patients,) reported that they were able to act after the surgery as independently as they had done before. Two patients stated that even before the injury and surgical intervention they were not able to cope independently with everyday life. The results show that the surgical treatment had not caused any disadvantage for the patients in terms of acting independently (Table 4). During the inpatient stay, complications such as pneumonia or uncomplicated urinary tract infections occurred in approximately 47% of cases (n = 39), which could be cured by antibiotic therapy. Screw malposition occurred in 2.41% (n = 2) of cases. Furthermore, no death was reported during the first year after the surgery.

### Calculation of the correlation of the Majeed score with the SF-36 score

The Pearson product-moment correlation coefficient, as a measure of the strength of the linear relationship between two variables, was applied to the sets of data sets summarized in Tables 5 and 6. It shows that all aspects of the analysis of patients’ collective are directly correlated with the Majeed score and the SF-36 score. The correlation coefficient values of the patients with surgical treatment were in the high category PF (r = 0.826), MH (r = 0.656), EWB (r = 0.756), VIT (r = 0.753), RP (r = 0.653), BP (r = 0.828), GH (r = 0.591), RE (r = 0.085) and SF (r = 0.043) for the nine different aspects outlined above. Thus, there is a positive correlation between the measurements of two different characteristics, i.e. the two different questionnaires using the Majeed score and the SF-36 score, respectively.

**Table 5:** Majeed score results

Majeed score [points]	Numbers (%)
≥ 85 Excellent	68 (81.93)
70–84 Good	7 (8.43)
55–69 Fair	4 (4.82)
≤ 55 Poor	4 (4.82)
n (total)	83

**Table 6:** SF-36 score results for the populations

SF-36 Domains	Total results of the patients (%) n = 83	Results of female patients (%) n = 51	Results of male patients (%) n = 32
Bodily pain (BP)	92.2	92.5	91.7
Mental Health (MH)	61.3	60.7	62.3
Social functioning & vitality (VIT)	69.6	70.4	68.3
Emotional well-being (EWB)	79.2	80.1	77.8
General health perception (GH)	63.0	61.8	64.8
Physical function (PF)	85.6	85.7	85.5
Social functioning (SF)	92.0	92.6	91.0
Role limitations due to physical health problems (RP)	90.7	90.7	90.6
Role limitations due to personal or emotional problems (RE)	92.7	94.0	90.6

## Discussion

Fragility fractures of the pelvis are often caused by low-energy trauma, in particularly in older patients [1]. In some cases, these injuries occur without any history of trauma. Osteoporosis is a common finding in the majority of these patients. It explains the present gender distribution of the patients, indicating that postmenopausal women are more affected by fragility fractures than men (see Table 2). This observation is also in line with the current report by Rommens et al. [7], describing that more than two-thirds of the patients with fragility fractures were females. Fragility fractures are traditionally treated conservatively. This treatment includes a period of bedrest and immobility which puts patients at risk of both severe early and long-term complications [3, 4]. According to Maier et al. [4], the outcome of a conservative therapy is poor, i.e. it is frequently associated with loss of social and physical independence, autonomy and a high mortality rate. Percutaneous insertion of sacroiliac screws is a surgical method to stabilize fractures in the area of the posterior pelvic ring. Recently, it had been shown that an anatomic reduction of the pelvic ring positively affects the long-term well-being of the patients [11]. In the cases of fragility fractures of the posterior pelvic ring, anatomical reduction is now less important, as there is often no major displacement. Rather, it is about stabilizing the posterior pelvic ring in order to relieve the patients’ pain. If a decision to undertake surgical treatment is instigated it is important to avoid any complications. The quality of life and functional outcome also need to be considered when performing surgery on these elderly patients with multiple co morbidities and potential osteoporosis. In the present study, we did not experience any long-term complications such as death or loss of independence during an observation period of at least one-year post surgery. We only observed low-grade complications such as pneumonia or uncomplicated urinary tract infection among the elderly patient population in approximately 47% of the cases (n = 39) during the inpatient stay, and they were cured by an antibiotic therapy. A complication rate of 47% appears to be high. However, it includes also low-grade complications such as the most common hospital-acquired infections which were treated with antibiotics without causing additional complications. It implies that patients need to be mobilized as early as possible, since the mortality rate among patients can increase significantly if they are immobilized in bed for an increased period of time without mobilization. After percutaneous screw fixation 2.41% (n = 2) of the patients needed a revision due to screw malposition. However, this rather small intervention did not affect a high scoring of the patients, showing that they achieved a good quality of life. Long-term complications associated with the conservative treatment [3] are overcome by the percutaneous screw fixation which results in functionally restoring the fractured posterior pelvic ring and lead to a long-term well-being of the patients [12].

The overall quality of life assessment after medical treatment is a subjective but relevant issue for the patient which can be critically judged using the Majeed score [9] in combination with the Short-Form Health Survey (SF-36) [10]. The SF-36 allows to interpret health aspects which include the physical and psychological status of the patient. We used this method to assess the health status of patients [13, 14], although it contains no specific reference values for pelvic fractures. We complemented therefore the SF-36 with the Majeed score covering the pelvic-specific questions [9] with the pre-casted questionnaire used in the present study. This combination allows not only to individually score the pain assessment of the SF-36 questionnaire, but adds additional important parameters relevant for judging the well-being of the patients. In accordance with the results of the SF-36 self-assessment of the patients, about 82% of the patients evaluated their pelvis injury-related health status positively, i.e. as an excellent outcome or good outcome (8%) of the medical treatment, and 10% were less positive with respect to their health condition and declared only fair or poor results. Best results (> 90%) were reported with respect to role limitations due to personal or emotional problems (RE), role limitations due to physical health problems (RP), bodily pain (BP) and social functioning (SF). Lower but still good scores were provided in the areas of vitality (VIT), physical functioning (PF), emotional well-being (EWB), general health perceptions (GH) as well as general mental health (MH) (Table 6).

Earlier studies, which did not use the Majeed score and evaluated only eleven patients led to similar results [15, 16]. Sanders et al. [15] examined their patients with the Oswestry Low Back Pain Disability Questionnaire, which is a scoring system for lower back pain, and with the Visual Analog Scale (VAS) which assesses mainly subjective complaints. Mehling et al. [16] studied their patients with a Pelvic Outcome score system without providing detailed information about the underlying procedure, in combination with a subjective pain follow up over a period of one year. Nevertheless, both studies show that the patients have clearly benefited from the surgical treatment, whereas studies on conservative treatment of patients with fragility fractures of the posterior pelvic ring report significantly increased complications including mortality and loss of independence in everyday life, i.e. 19% of the patients died during the first year after the fracture and 27% of the patients had to move into nursing homes [3]. This study used the timed up-and-go-test to identify possible restrictions on mobility, and the Visual Analog Scale (VAS) to assess pain during movement and rest. Additionally, the patients were examined by score systems for assessing comorbidities (using the Charlson Comorbidity Index (CCI) and American Society of Anesthesiologists (ASA)-score). The use of these methods shows that surgical treatment by percutaneous screw fixation can preserve the functional capacity and thereby support social independence in elderly patients suffering from low-energy pelvic ring fractures. Our study confirms these recent results and includes additional aspects such as social functioning, vitality, general mental health and health perception as well as the mental health of the patients, all of which were positively scored by the patients. Furthermore, since the Majeed scores and the SF-36 results can be directly and positively correlated with respect to the subjectively felt well-being of the patients, the SF-36 questionnaire, although it does not address specific pelvic-related questions, can indeed be used to assess the success of the surgery of patients which experienced pelvic injuries. No obvious difference with respect to the subjective well-being was observed between patients which belong to different FFP classification groups. This finding suggests that surgical treatment with percutaneous sacroiliac screws is indeed favorable for all FFP-patients.

Furthermore, the NRS value, which quantified the subjective tenderness-related pain sensation at the sacroiliac joint of the patients, showed a very low value with an average of 1.60 ± 2.14 points which refers to a low subjective pain level postoperatively. Thus, the outcome of the present study allows the conclusion that surgical treatment as described here has a beneficial impact on the quality of life of older patients who suffered from a fragility fracture of the posterior pelvic ring.

It should be noted that our study has limitations. The Majeed score includes, among others, the aspects work and sexuality, and these aspects of life and well-being are difficult to evaluate in older patients. Another limitation of our study is the fact that in addition to the posterior pelvic ring the anterior pelvic ring was also injured in some cases (n = 35), be it as a fracture or an injury to the symphysis, of the os ischia or the os pubis. In the cases where a stable fracture situation could be established by percutaneous screw osteosynthesis, the anterior pelvic ring was not surgically addressed. However, if the fracture remained unstable, the anterior pelvic ring was additionally stabilized by implanting an external fixator. Our study focused on the posterior pelvic ring only. In addition, possible comorbidities of the patients such as smoking, diabetes and osteoporosis were not included as separate parameters in the study. Furthermore, the patients were not examined with respect to years after the surgery event, since the corresponding numbers of patients to be interviewed would not be suitable to provide a meaningful statistical analysis. Finally, we did not perform a comparative study with conservatively treated patients, and used published data for comparison instead. Since our results suggest a beneficial clinical outcome for patients which received surgical treatment, we plan a comparative randomized large-scale study to both confirm and extend our current results.

## Conclusion

Operative treatment of patients with fragility fractures of the posterior pelvic ring, as described here, has a measurable positive impact on the well-being of patients when compared to conservative treatment without surgery. Whereas conservative treatment results both in 19% morbidity during the first year after the fracture [3] and an increase of sustainable pain [3, 4], such cases were not observed after surgery as revealed by the patients examined in our study. Thus, it appears that fixation of the fractures inhibits or even prevents early morbidity after the fracture and prevents pain as reported for patients that were treated conservatively. In addition, also general health perceptions, i.e. physical functioning, vitality, mental health, social functioning and emotional well-being, were not affected since patients reported that they lived up to the level prior to their accident. Therefore, surgical treatment of patients with fragility fractures as described here can add positively to the clinical outcome and should be considered.

## Data Availability

The datasets supporting the conclusions of this article are included within the article (and its additional files). Additional datasets used for the current study are available from the corresponding author upon request.

## Abbreviations

ASA: American Society of Anesthesiologists
BP: Bodily pain
CCI: Charlson Comorbidity Index
CT: Computer tomography
EWB: Emotional well-being
FFP: Fragility fractures of the pelvis
GH: General health perceptions
ICD: International classification of diseases
MH: General mental health
NRS: Numerical rating scale
OPS: Operations and procedures key
PF: Physical functioning
RE: Role limitations due to personal or emotional problems
RP: Role limitations due to physical health problems
SF: Social functioning
SF-36: Short Form Healthy Survey-36 score
VIT: Social functioning, energy/fatigue or vitality
